# Supplementary figures and images for: Transcriptional regulation in the absence of inositol trisphosphate receptor calcium signaling
Source: Front Cell Dev Biol. 2024 Dec 6;12:1473210. doi: 10.3389/fcell.2024.1473210 (PMC11659226; doi:10.3389/fcell.2024.1473210)

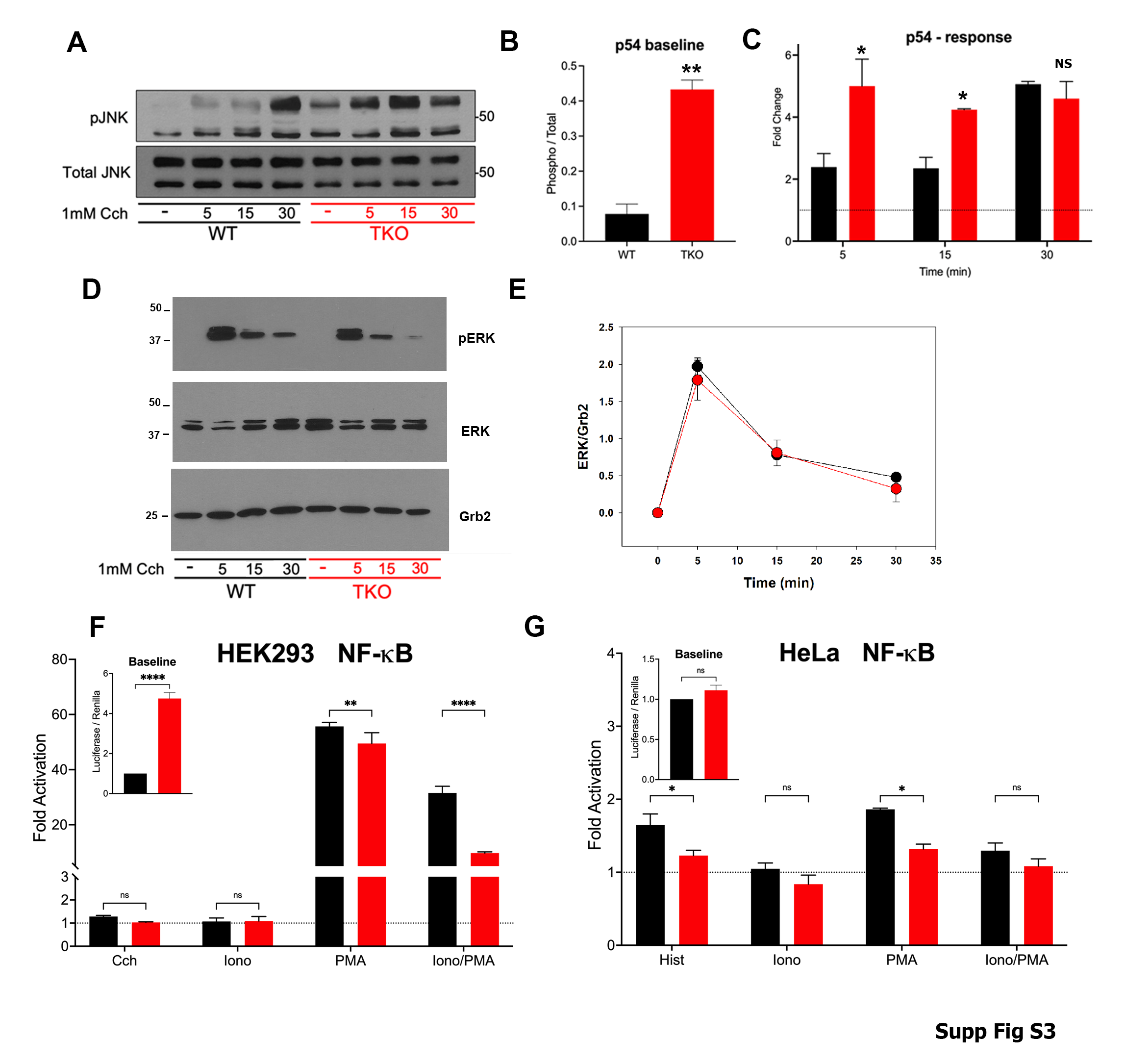

Supplement: Supplementary file 1 [file Image3.tif]

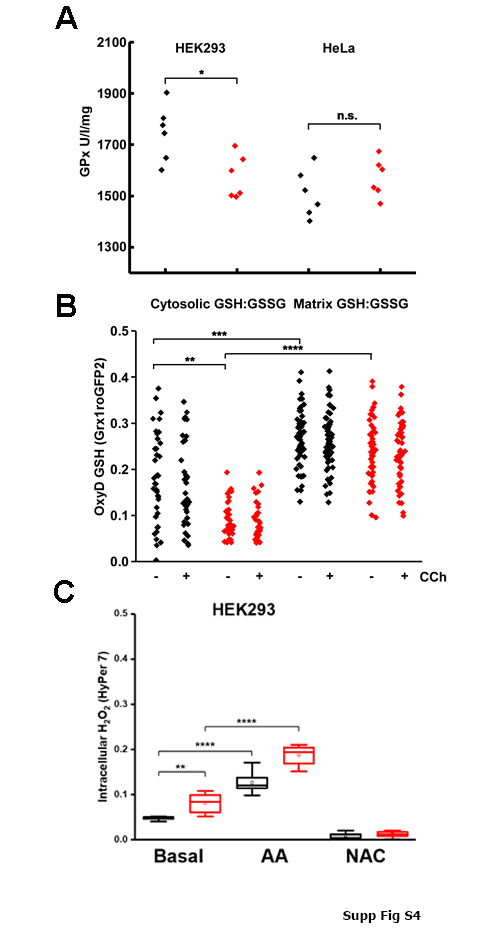

Supplement: Supplementary file 2 [file Image4.tif]

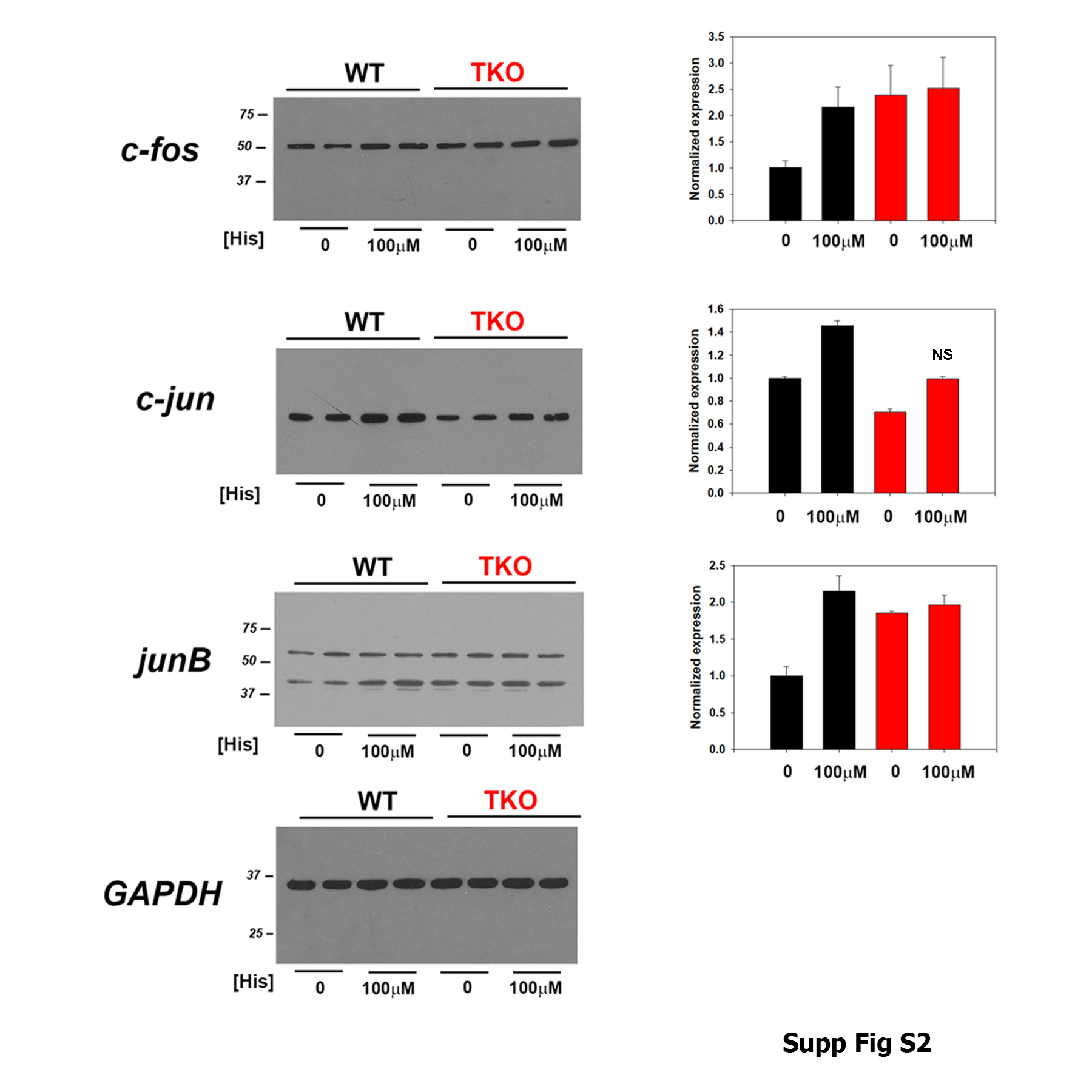

Supplement: Supplementary file 3 [file Image2.tif]

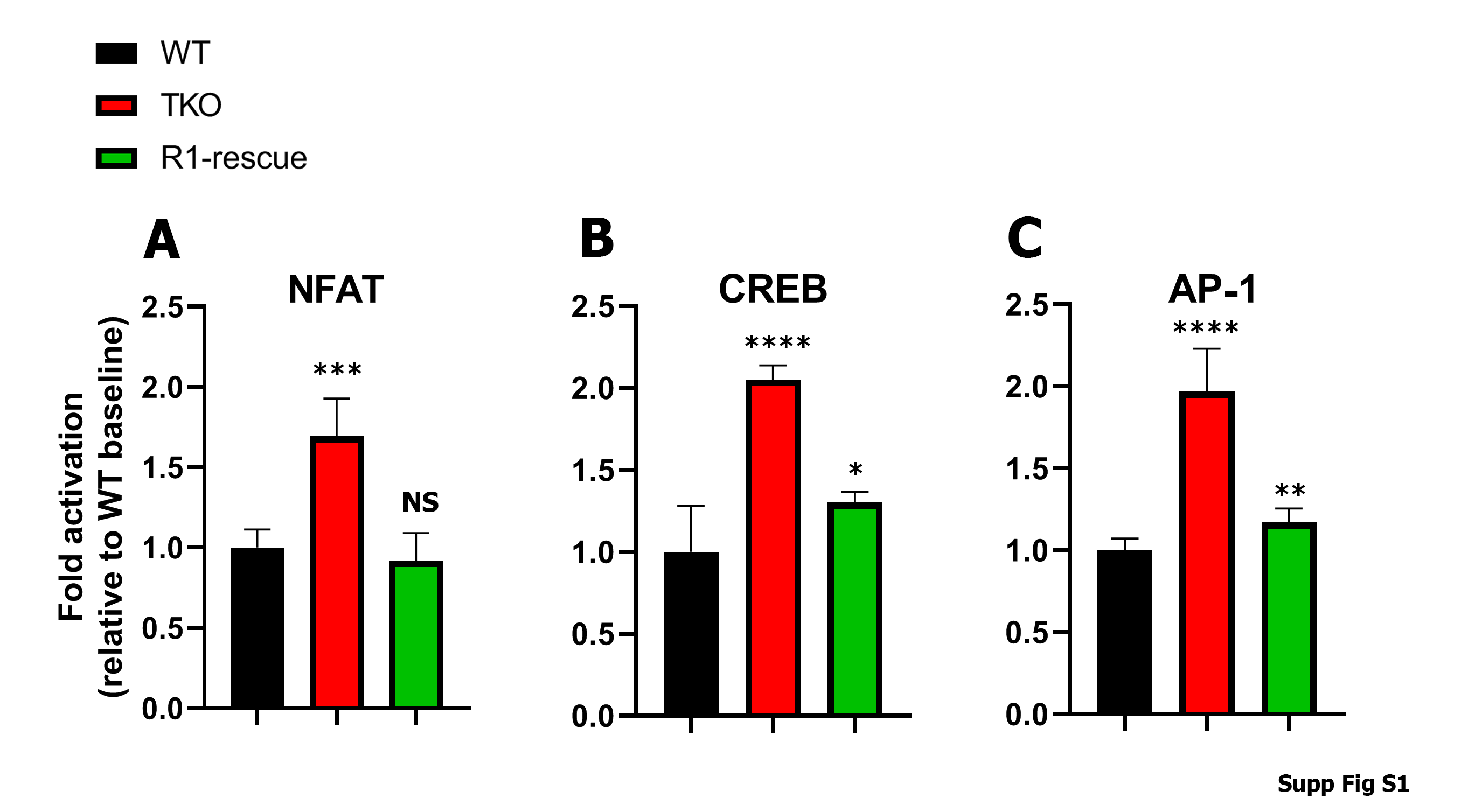

Supplement: Supplementary file 4 [file Image1.tif]

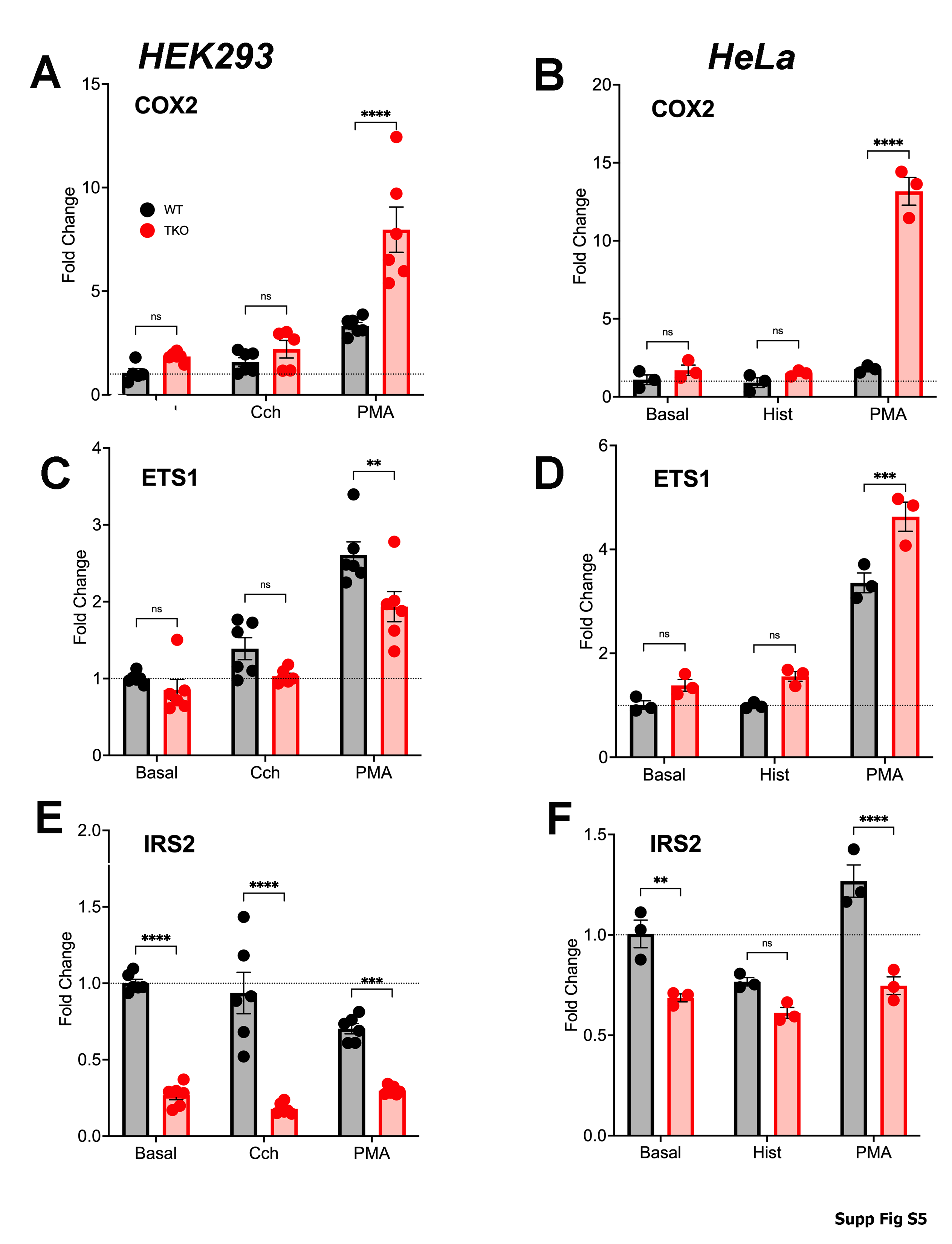

Supplement: Supplementary file 5 [file Image5.tif]
